# Immune Suppression by Neutrophils in HIV-1 Infection: Role of PD-L1/PD-1 Pathway

**DOI:** 10.1371/journal.ppat.1003993

**Published:** 2014-03-13

**Authors:** Nathan L. Bowers, E. Scott Helton, Richard P. H. Huijbregts, Paul A. Goepfert, Sonya L. Heath, Zdenek Hel

**Affiliations:** 1 Department of Pathology, University of Alabama at Birmingham, Birmingham, Alabama, United States of America; 2 Center for AIDS Research, University of Alabama at Birmingham, Birmingham, Alabama, United States of America; 3 Department of Medicine, University of Alabama at Birmingham, Birmingham, Alabama, United States of America; 4 Department of Microbiology, University of Alabama at Birmingham, Birmingham, Alabama, United States of America; National Institutes of Health, United States of America

## Abstract

HIV-1 infection is associated with a progressive loss of T cell functional capacity and reduced responsiveness to antigenic stimuli. The mechanisms underlying T cell dysfunction in HIV-1/AIDS are not completely understood. Multiple studies have shown that binding of program death ligand 1 (PD-L1) on the surface of monocytes and dendritic cells to PD-1 on T cells negatively regulates T cell function. Here we show that neutrophils in the blood of HIV-1-infected individuals express high levels of PD-L1. PD-L1 is induced by HIV-1 virions, TLR-7/8 ligand, bacterial lipopolysaccharide (LPS), and IFNα. Neutrophil PD-L1 levels correlate with the expression of PD-1 and CD57 on CD4^+^ and CD8^+^ T cells, elevated levels of neutrophil degranulation markers in plasma, and increased frequency of low density neutrophils (LDNs) expressing the phenotype of granulocytic myeloid-derived suppressor cells (G-MDSCs). Neutrophils purified from the blood of HIV-1-infected patients suppress T cell function via several mechanisms including PD-L1/PD-1 interaction and production of reactive oxygen species (ROS). Collectively, the accumulated data suggest that chronic HIV-1 infection results in an induction of immunosuppressive activity of neutrophils characterized by high expression of PD-L1 and an inhibitory effect on T cell function.

## Author Summary

Despite 30 years of intensive research, our understanding of how HIV-1 virus undermines the ability of the immune system to fight common infections is limited. Although we know that T cells, a key cell population that normally fights invading pathogens, lose their ability to function in HIV-1-infected individuals, we do not fully understand why. Here, we found that HIV-1 virus activates another type of cells, neutrophils, the most common type of white cell in the blood. Activated neutrophils negatively affect the function of T cells and prevent them from producing cytokines, protective proteins that serve as messengers orchestrating the immune response to bacteria and viruses. This newly identified mechanism of immune suppression mediated by neutrophils may alter our understanding of HIV-1 pathogenesis and result in a design of novel therapies targeting the loss of immune function in HIV-1/AIDS.

## Introduction

Neutrophils, the most abundant leukocyte population, are traditionally recognized as essential effector cells of the innate immune system in the host defense against invading pathogens [1]. In recent years, a new appreciation of the role of neutrophils in interacting with and regulating the adaptive arm of the immune system has emerged [1], [2]. Neutrophils co-localize and actively communicate with T cells at sites of infection and migrate to the draining lymph nodes where they are involved in the induction and regulation of cellular and humoral immune responses by exerting pro-inflammatory or anti-inflammatory function [2]–[4]. Accumulating evidence supports the role played by neutrophils in the negative regulation of T cell function via production of reactive oxygen species (ROS) and arginase-1 [2], [5]–[7]. A recent study has identified an immunosuppressive population of CD16^+^CD62L^low^ neutrophils that is induced in human volunteers following injection of a low dose of bacterial lipopolysaccharide and inhibits T cell function by local release of hydrogen peroxide into the immunological synapse between the neutrophil and T cell [7].

A population of cells referred to as myeloid-derived suppressor cells (MDSCs) has been identified in peripheral blood mononuclear cells (PBMCs) in multiple pathological conditions involving inflammation including cancer, chronic bacterial and viral infection, trauma, and sepsis [6], [8]. MDSCs have been shown to serve as a negative feedback mechanism preventing potential damage caused by acute and chronic inflammation. Data recently obtained in sepsis, chronic inflammatory conditions and several types of cancers demonstrate the presence of a population of MDSCs of granulocytic origin (G-MDSCs). G-MDSCs likely originate from circulating neutrophils that acquire low density neutrophil (LDN) phenotype and co-segregate in the PBMC fraction on a density gradient [6], [8]–[10]. It is unclear at present whether LDN/G-MDSCs originate by granulopoiesis from dedicated suppressive progenitors in the bone marrow or whether they represent a functional subset of neutrophils that acquired the immunosuppressive phenotype in response to specific signals in the periphery [6]. G-MDSCs display a remarkable ability to suppress T cell-mediated immune responses by multiple mechanisms including release of arginase-1 resulting in a depletion of arginine and downregulation of TCR ζ chain, production of reactive oxygen species (ROS), production of regulatory cytokines, and induction of regulatory T cells [6], [8].

CD8^+^ and CD4^+^ T cells play a key role in controlling HIV-1 replication and progression to AIDS. However, HIV-1 infection is associated with a progressive loss of T cell functional capacity including decreased responsiveness to antigenic stimuli, lowered capacity to produce cytokines, and reduced proliferative and cytotoxic activity [11]–[15]. Loss of CD4^+^ T cells and functional impairment of HIV-1-specific CD8^+^ and CD4^+^ T cells eventually results in a failure of host immune system to maintain control of HIV-1 leading to an accelerated disease progression. HIV-1-specific T cells from rapidly progressing patients exert decreased cytotoxic and proliferative activity and produce reduced levels of TNFα, IL-2, IFNγ, and CD107a compared to T cells from non-progressors [12], [13]. T cell exhaustion in HIV-1 infection is associated with increased expression of programmed death-1 (PD-1) and CD57 on the surface of CD4^+^ and CD8^+^ T cells [14]–[17]. Binding of PD-1 on T cells to the inhibitory ligand PD-L1 expressed on cells of myeloid lineage including myeloid dendritic cells (DCs), monocytes, and macrophages negatively regulates T cell proliferation and production of effector cytokines [18]–[21]. In simian immunodeficiency virus (SIV)-infected rhesus macaques, high PD-1 expression is associated with an impaired response of SIV-specific T cells during both acute and chronic infection. Importantly, blocking of PD-L1/PD-1 axis *in vivo* in chronic SIV infection restores the function of SIV-specific cellular and humoral immune responses, improves viral control, and reduces immune activation [22]–[24]. These observations support a critical role of PD-L1/PD-1 interaction in the regulation of immune environment in HIV-1 infection and serve as a basis for ongoing clinical trials assessing the therapeutic potential of blocking PD-L1/PD-1 signaling in chronically infected individuals.

Here we demonstrate that neutrophils in the blood HIV-1-infected individuals express high levels of surface PD-L1 and suppress the function of T cells via ROS and PD-L1/PD-1 pathways. The upregulated expression of PD-L1 on neutrophils correlates with the presence of LDNs that co-segregate in the mononuclear cell fraction on a density gradient and express G-MDSC phenotype. Neutrophil PD-L1 is induced directly by HIV-1 virions, IFNα, TLR-7/8 ligand R848, and LPS. The presented data suggest that the induction of PD-L1 on neutrophils, the most abundant leukocyte population, in concert with high PD-1 expression on T cells significantly contributes to the ongoing T cell exhaustion and immune suppression in HIV-1 infection.

## Results

### Neutrophils from HIV-1-infected individuals express elevated levels of PD-L1

**Figure 1:**
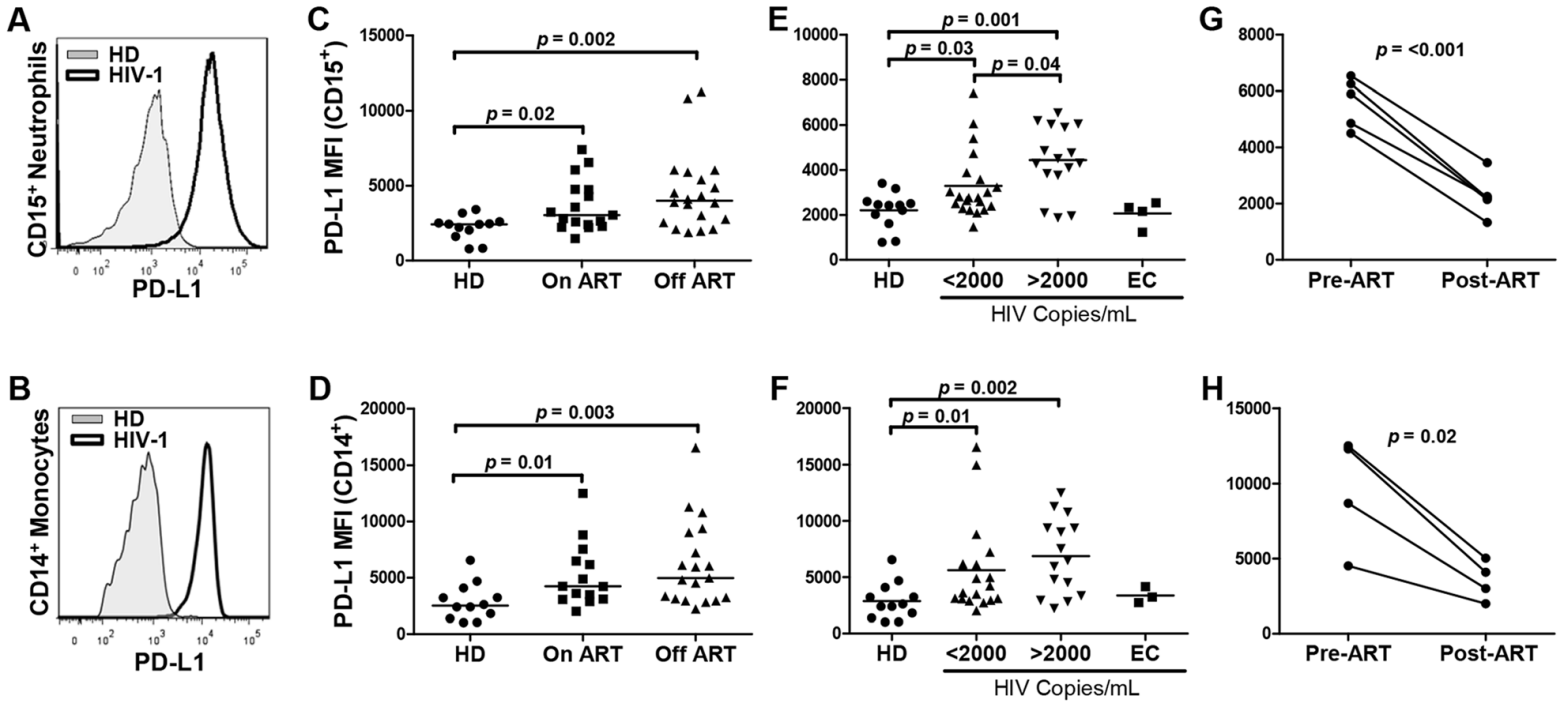
Neutrophils from HIV-1 infected individuals express elevated levels of surface PD-L1. A, B) Representative histogram of PD-L1 expression on CD15^+^ neutrophils (A) and CD14^+^ monocytes (B) in fresh blood obtained from a healthy donor (HD) and HIV-1-infected individual. C, D) PD-L1 expression on CD15^+^ neutrophils (C) and CD14^+^ monocytes (D) of HDs (N = 12), HIV-1-infected patients on ART (N = 17), and subjects not treated with ART (off ART; N = 20). MFI, mean fluorescent intensity. E, F) PD-L1 expression on CD15^+^ neutrophils (E) and CD14^+^ monocytes (F) of HD (N = 12) and HIV-1-infected individuals with viral loads <2,000 vRNA copies/ml of plasma (N = 21), >2,000 vRNA copies/ml of plasma(N = 19), or elite controllers (EC: <50 copies/ml; off ART; N = 4). Statistical analysis on C-F was performed using the Mann Whitney rank sum test. G, H) PD-L1 expression on CD15^+^ neutrophils (G) (N = 5) and CD14^+^ monocytes (H) (N = 4) of HIV-1-infected individuals prior to and following the administration of ART.

To characterize the phenotype of neutrophils in freshly obtained blood of HIV-1-infected individuals, multiparameter flow cytometry analysis of SSC^high^ CD15^+^ CD33^+/dim^ CD11b^+^ neutrophil population was performed to determine the levels of surface markers including CD11b, CD15, CD16, CD33, CD80, CD86, CD115 (M-CSFR), HLA-DR, and PD-L2 (Figure S1). No significant differences in the levels of expression of these markers were observed between HIV-1 patients and uninfected controls. In contrast, circulating neutrophils from HIV-1-infected patients expressed significantly elevated levels of PD-L1 compared to neutrophils from healthy uninfected donors, irrespective of antiretroviral therapy (ART) status (*p = *0.02 and 0.002 in individuals on and off ART, respectively; Fig. 1A, C). Neutrophil PD-L1 expression was significantly higher in patients with HIV-1 viral load >2,000 copies of viral RNA (vRNA) per ml of plasma compared to patients that successfully controlled viral replication (*p* = 0.04; Fig. 1E). No increase in PD-L1 expression was observed in elite controllers (EC) restricting viral proliferation below 50 vRNA copies/ml in an absence of ART. The extent of the increase of the neutrophil PD-L1 expression was comparable to the increase observed on CD14^+^ monocytes from HIV-1-infected individuals (Fig. 1B,D,F), as published previously [25], [26]. The analysis revealed a trend to a direct correlation between HIV-1 viral load and PD-L1 expression on blood neutrophil population; however, the trend has not reached statistical significance (*p* = 0.1). To address whether neutrophil PD-L1 expression is modulated by ART, fresh blood from 5 HIV-1-infected subjects was analyzed before and after the initiation of ART resulting in a successful control of HIV-1 replication. PD-L1 expression was significantly reduced following ART implementation on both neutrophil and monocyte populations (Figure 1G, H).

### Neutrophils from HIV-1-infected subjects suppress T cell function in part via the PD-L1/PD-1 pathway

**Figure 2:**
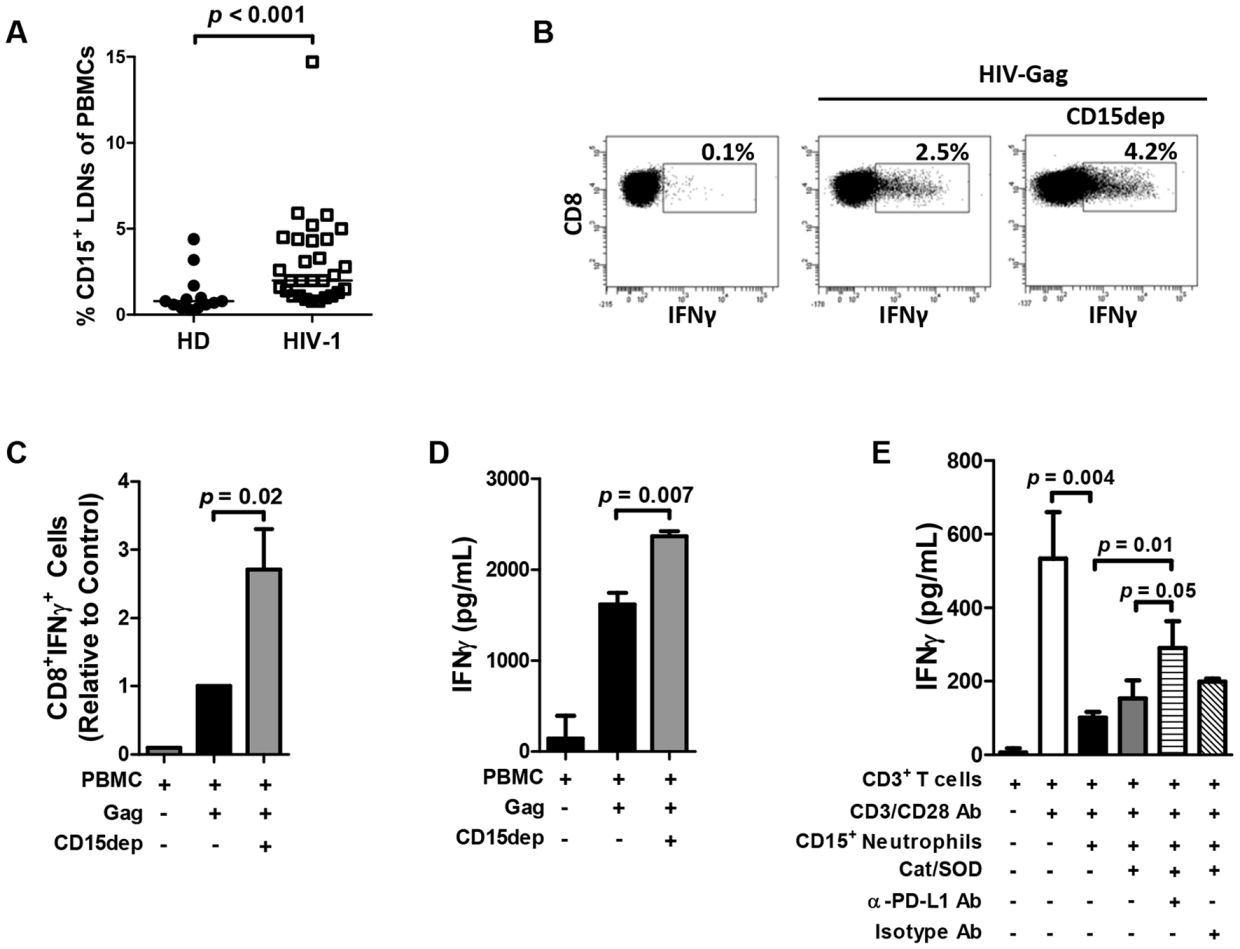
Neutrophil suppression of T cell function is mediated in part via the PD-L1/PD-1 pathway. A) PBMCs of HIV-1-infected individuals display higher frequency of CD15*^+^* LDNs compared to healthy donors (N = 13 and 29). Statistical analysis was performed using the Mann Whitney rank sum test. B) Depletion of CD15^+^ neutrophils co-segregating with the PBMC fraction results in an enhanced frequency of antigen-specific IFNγ-producing cells. Representative intracellular cytokine staining of CD8^+^ T cells from HIV-1-positive subject. PBMCs or CD15^+^ cell-depleted PBMCs were incubated with HIV-1-Gag peptide pool for 24 hrs. C) Summary of normalized data of four experiments using independent HIV-1-infected donors. Mann Whitney rank sum test. D) Depletion of CD15^+^ cells from PBMCs results in an increase of IFNγ production in culture supernatant in response to stimulation with HIV-1 Gag (N = 3). E) CD3^+^ T cells were isolated from HIV-1-infected subjects with high PD-L1 expression and stimulated with α-CD3 and α-CD28 antibodies. Neutrophils were incubated with activated T cells at a 5∶1 ratio in the presence of anti-PD-L1 blocking antibody or isotype control for 24 hours. The experiment was performed in the presence of ROS scavengers SOD and catalase. IFNγ release into media was determined by ELISA. A representative experiment of three independent experiments using separate HIV-1-infected donors is presented; statistical significance was analyzed using Student's *t*-test.

Previous studies have identified a population of suppressive neutrophils that acquire the phenotype of low-density neutrophils (LDN), co-segregate with PBMCs on a density gradient, and display the phenotype of G-MDSCs [6], [8]–[10], [27]. Here we show that HIV-1-infected individuals display higher frequency of LDNs in PBMCs compared to healthy donors (*p*<0.001; Fig. 2A). Importantly, elevated PD-L1 expression on neutrophils in whole blood correlates with the frequency of CD15^+^ LDNs in PBMCs (R = 0.6; *p = *0.01; data not shown). Surface expression of PD-L1 on LDNs was not significantly different from that on blood neutrophils. LDNs expressed elevated levels of CD15, CD33, and CD66b and lower levels of CD62L, CD80, CD114, and CXCR4 compared to whole blood neutrophils (N.B., Z.H., unpublished data) [28]. Depletion of CD15^+^ LDN cells from PBMCs of HIV-1-infected donors resulted in an increase in the percentage CD8^+^IFNγ^+^ T cells (Fig. 2B,C; *p* = 0.02), a trend towards an increase in the frequency of CD4^+^IFNγ^+^ T cells (3-fold mean increase; *p* = 0.6), and an increase in IFNγ production (Fig. 2D; *p* = 0.007) in response to stimulation with HIV-1 Gag overlapping peptide pool. Similar results were obtained following non-specific stimulation with PHA or microbeads coated with antibodies against CD3 and CD28 antigens suggesting that the LDN-mediated inhibition is independent of specific antigen presentation [6], [7], [10].

Analysis of freshly prepared PBMCs from HIV-1-infected individuals demonstrated elevated levels of staining with DCF-DA in LDNs, an indicator of ROS production, compared to uninfected volunteers (*p* = 0.008) and a significant correlation between DCF-DA staining and elevated levels of PD-L1 on neutrophils (R = 0.9; *p* = 0.01; data not shown). It has been previously demonstrated that the production of ROS represents a major mechanism of neutrophil and LDN/G-MDSC –mediated suppression of T cell function [6], [7]. Since the PD-L1 on the surface of monocytes, DCs, and other cells of myeloid lineage negatively regulates T cell proliferation and production of effector cytokines [18]–[21], we hypothesized that direct PD-L1/PD-1 interaction may contribute to neutrophil-mediated suppression of T cell function. To address the specific contribution of PD-L1 to neutrophil-mediated T cell suppression, polymorphonuclear cells (PMNs) purified from the blood of HIV-1-infected patients were depleted of any residual CD14^+^ monocytic cells and incubated for 24 hours with CD3/CD28-activated autologous T cells at 5∶1 ratio resulting in about 70% decrease in IFNγ production (Fig. 2E). Importantly, the inhibition of IFNγ production was partially reversed in the presence of ROS scavengers in combination with antibodies blocking PD-L1 (*p* = 0.05) but not control isotype antibodies. Reversal of inhibition by blocking PD-L1/PD-1 interaction was contingent on elevated PD-1 expression on T cells and PD-L1 expression on neutrophils (MFI >4,500; three independent experiments using separate donors). PD-L1 blocking had little effect on neutrophils (PMNs) from HIV-1-infected patients or uninfected volunteers with low PD-L1 expression (MFI <3,000).

### Neutrophil PD-L1 expression is induced by HIV-1, IFNα, TLR-7/8 ligand, and bacterial lipopolysaccharide

**Figure 3:**
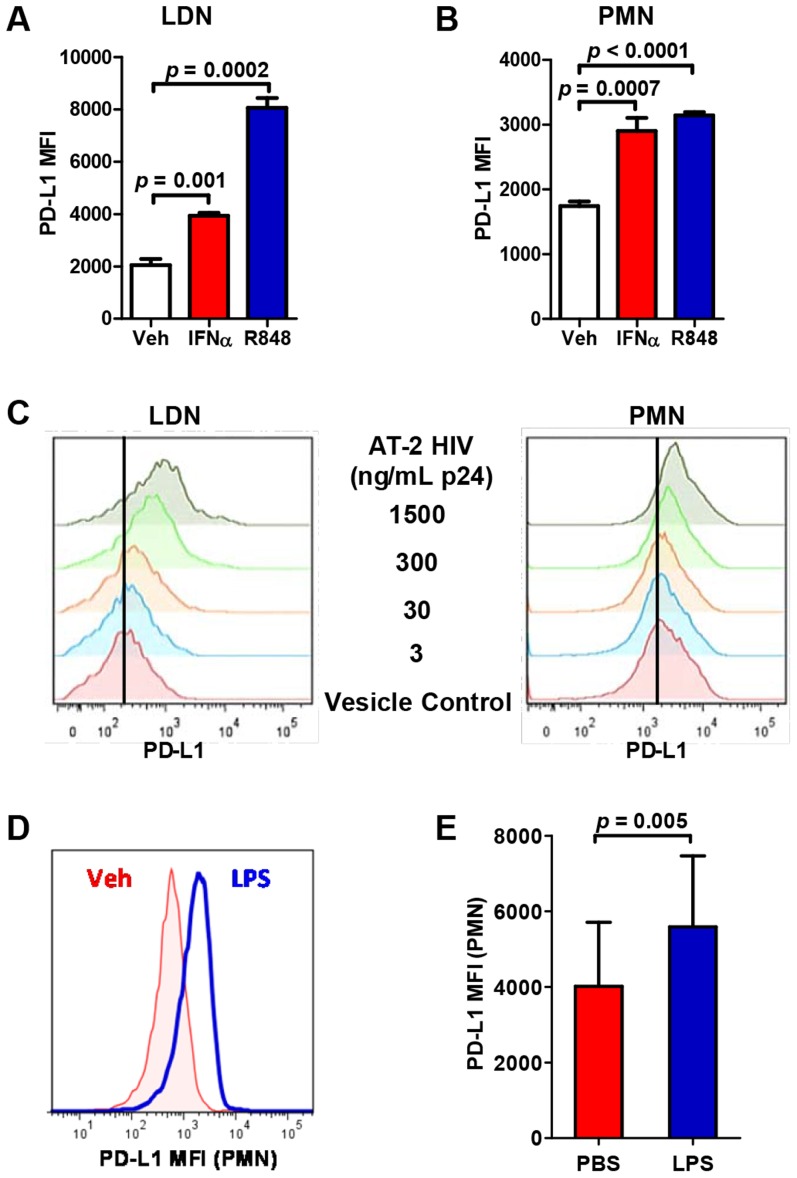
PD-L1 expression on neutrophils is induced by HIV-1 virions, IFNα, TLR-7 ligand R848, and LPS. A, B) PBMCs or PMNs were stimulated with IFNα (1,000 U/ml), R848 (5 µg/ml), or vehicle (Veh) for 24 hours and PD-L1 expression on CD15^+^ LDNs and PMNs was determined by flow cytometry. Representative of three separate experiments using separate donors; statistical significance was analyzed using Student's *t*-test. C) PBMCs or PMNs were incubated with AT-2-inactivated HIV virions (AT-2 HIV) at indicated doses or control microvesicles from untransfected cell cultures for 24 hours. D) Representative histogram of PD-L1 expression on PMNs incubated for 24 hrs with LPS (100 ng/mL). E) Summary of PD-L1 expression on PMNs in the presence or absence of LPS (N = 3); statistical significance was analyzed using Student's *t*-test.

The signaling events leading to increased PD-L1 expression on myeloid cells in HIV-1 infection are not completely understood. PD-L1 expression on monocytes and plasmocytoid dendritic cells (pDCs) was shown to be directly induced by HIV-1 virions and ligands of the TLR-7 and -8 receptors [25], [26]. Furthermore, recognition of the single-stranded RNA of the HIV-1 genome by TLR7 and 8 in pDCs results in a production of IFNα that directly induces PD-L1 expression on monocytes and other cell types [29]. We determined the effect of HIV-1 virions, TLR-7/8 ligand R848, and IFNα on PD-L1 expression on neutrophils. Stimulation with IFNα or TLR-7/8 ligand R848 results in a significantly increased expression of PD-L1 on LDNs that are present at low frequency in PBMCs of healthy donors as well as on purified CD15^+^ PMNs (>95% purity; Fig. 3A,B). To directly test the effect of HIV-1 virions on PD-L1 expression, PBMCs or purified PMNs from healthy donors were incubated with AT-2-inactivated HIV-1 MN virions or control microvesicle preparation absent of viral proteins or RNA. Neutrophil (LDN and PMN) expression of PD-L1 was increased in a dose-dependent manner in response to the treatment with AT-2 HIV-1 (Fig. 3C; *p* = 0.03 and 0.01 for CD15^+^ LDNs or PMNs, respectively). In addition, treatment with HIV-1 virions resulted in a decreased expression of CD62L on LDNs; other neutrophil surface proteins were not significantly modulated (Figure S2A). Relative induction (fold of increase) of PD-L1 expression following stimulation with R848 or AT2 HIV-1 was higher in LDNs than in purified PMNs; this may reflect a contributing effect of a factor or factors produced by other cell population. A direct correlation between neutrophil PD-L1 expression and IFNα concentration in plasma was detected in patients with <500 CD4^+^ T cells/ml of blood (R = 0.7; *p* = 0.04). To assess the potential contribution of IFNα to the induction of PD-L1, PBMCs from HIV-1-seronegative donors were cultured with R848 or HIV-1 in the presence of antibodies blocking the cellular receptor for IFNα (IFNAR). The presence of IFNAR-blocking antibody partially inhibited HIV-1- and R848- induced PD-L1 expression on whole blood neutrophils (indicating a partial contribution of IFNα to PD-L1 induction by TLR-7/8 ligands (*p* = 0.04 and 0.003, respectively; Figure S2B).

Translocation of LPS and other microbial products from gut lumen across the damaged intestinal epithelial barrier contributes to the systemic immune activation observed in HIV-1 infection [30]. Interaction between LPS, CD14, myeloid differentiation-2 (MD-2), and TLR-4 results in an activation of nuclear factor kappa-light-chain-enhancer of activated B cells (NFκB) signaling pathway and shedding of soluble CD14 (sCD14) from myeloid cells. Plasma levels of sCD14 indicate the degree of bacterial translocation and independently predict disease progression in HIV-1 patients [31]–[33]. In agreement with previously studies, we observed elevated levels of plasma sCD14 in the cohort of HIV-1-infected volunteers compared to healthy uninfected donors (*p*<0.001). Pillay *et al.* have recently observed that an injection of low dose of bacterial lipopolysaccharide in human volunteers induces a suppressive subpopulation of neutrophils [7]. We have therefore addressed the hypothesis that LPS directly modulates PD-L1 expression on neutrophils. PD-L1 expression on PMNs of healthy donors was significantly increased following the stimulation with LPS (Fig. 3D,E; *p* = 0.005) and reversed in the presence of polymyxin B (Figure S2C). Although higher concentration of LPS was used in an *in vitro* experiment than is typically found *in vivo*, the effect of LPS on neutrophils is enhanced *in vivo* by an interaction with LPS-binding protein [34]. This data suggests that bacterial translocation may contribute to the elevated PD-L1 expression on neutrophils of HIV-1-infected patients at sites of high local concentration of LPS. In addition, treatment with LPS resulted in an upregulation of DC-SIGN and down-regulation of CD16 but no modulation of CD62L (Figure S2A).

### PD-L1 expression on neutrophils correlates with markers of T cell exhaustion in HIV-1-infected individuals

**Figure 4:**
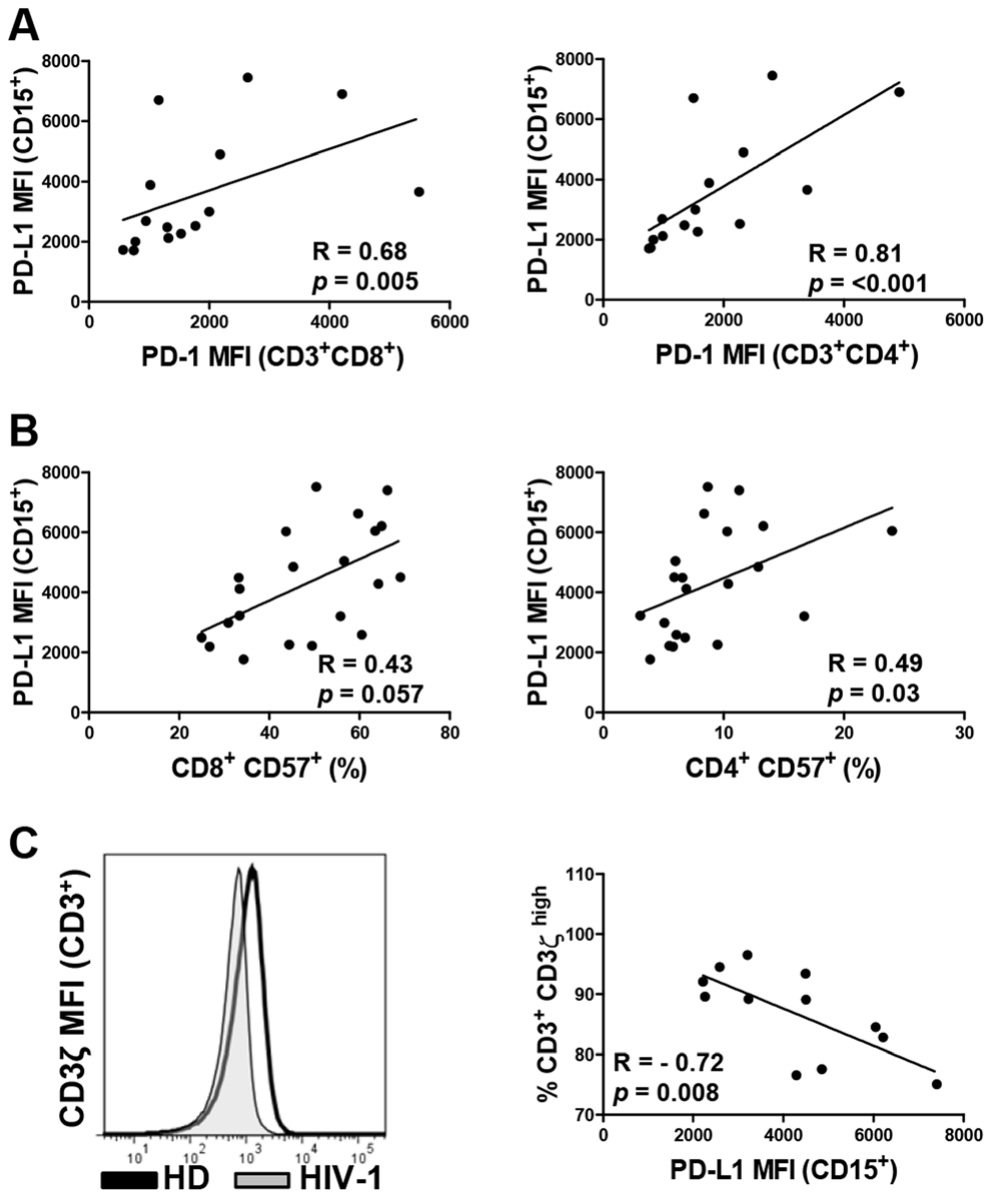
PD-L1 expression on neutrophils correlates directly with PD-1 and CD57 expression on T cells and inversely with the expression of CD3ζ chain on T cells. CD3^+^CD8^+^ and CD3^+^CD4^+^ T cells from PBMCs were stained for PD-1 (A), CD57 (B), and CD3ζ (C). A) Correlation between PD-1 expression on CD8^+^ and CD4^+^ T cells and PD-L1 expression on neutrophils in blood. B) Correlation between the percentage of CD57^+^ cells of total CD3^+^CD8^+^ and CD3^+^CD4^+^ T cells and PD-L1 expression on neutrophils in blood. C) Flow cytometry analysis of the expression of CD3ζ chain on T cells and correlation between the percentage of CD3^+^ CD3ζ ^high^ population and PD-L1 expression on neutrophils. Spearman's rank correlation coefficients (R) and *p* values are indicated for each correlation; lines represent linear regression analysis.

**Figure 5:**
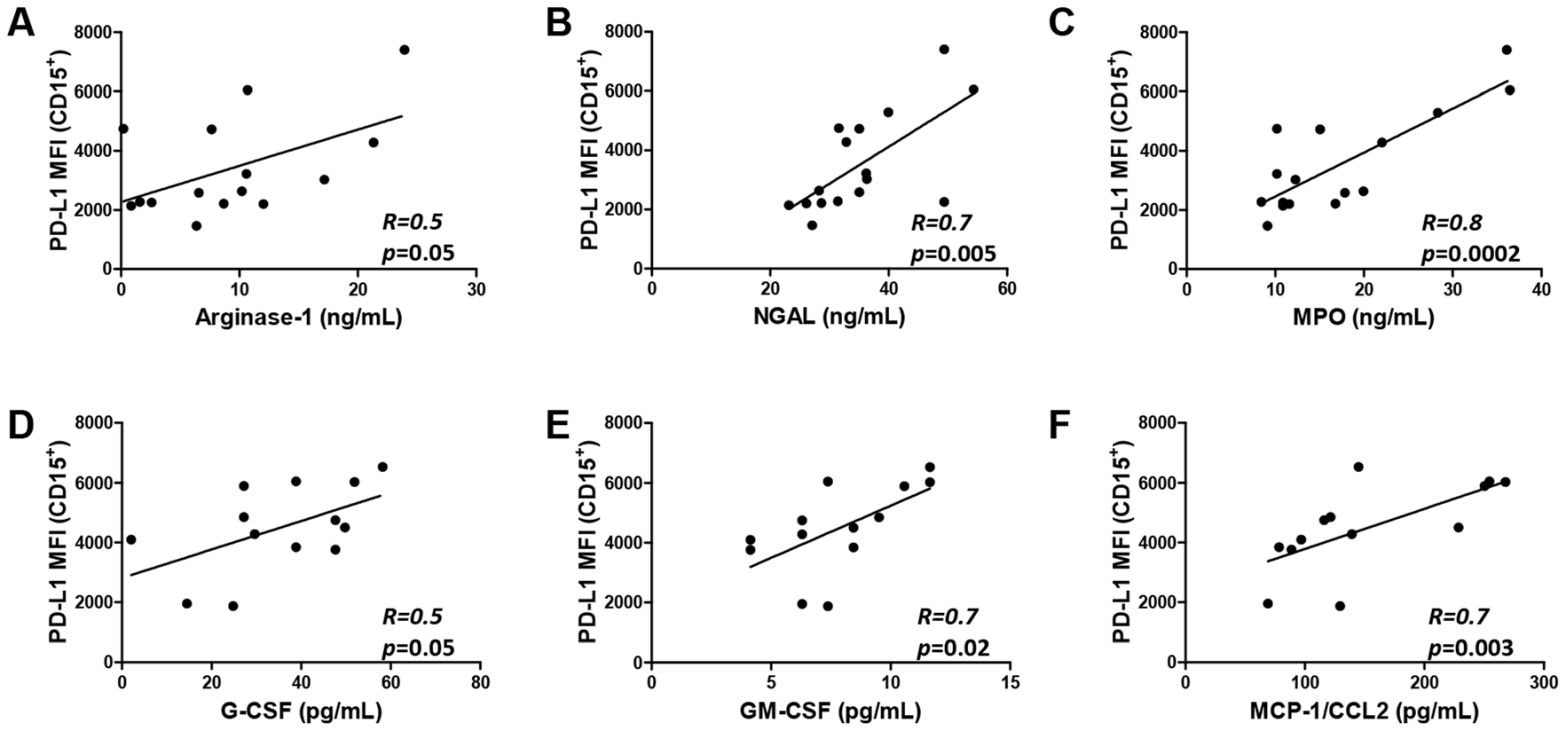
Neutrophil PD-L1 expression correlates with elevated levels of markers of neutrophil degranulation *in vivo*. A–C) PD-L1 expression on neutrophils correlates with plasma levels of arginase-1 (A), neutrophil gelatinase-associated lipocalin (NGAL) (B), and myeloperoxidase (MPO) (C) (N = 16). Analyzed by Pearson product-moment correlation test; D'Agostino & Pearson omnibus normality test of normal distribution of data passed for all populations (α = 0.05). D–F) PD-L1 expression on neutrophils correlates with plasma levels of G-CSF (D), GM-CSF (E), and MCP-1/CCL2 (F) in HIV-1-infected donors (N = 13).

T cell exhaustion in HIV-1 infection is associated with elevated expression of PD-1 on CD4^+^ and CD8^+^ T cells [16], [17]. CD57 is expressed primarily on T cells at late or terminal stages of differentiation and marks a state of replicative senescence characterized by a loss of the proliferative and target cell killing capacity [35], [36]. Here we show that PD-L1 expression on circulating neutrophils closely correlates with the expression of PD-1 on CD8^+^ and CD4^+^ T cells (Fig. 4A; *p = *0.005 and <0.001, respectively) and with the expression of CD57 on CD4^+^ T cells (Fig. 4B; *p = *0.03; gating strategy in Figure S3). Interestingly, correlations of PD-1 and CD57 expression on CD4^+^ and CD8^+^ T cells with PD-L1 expression on neutrophils was stronger than the respective correlations with PD-L1 levels on monocytes (Figure S4). Neutrophil-derived arginase-1 is known to down-regulate the CD3ζ chain via the depletion of L-arginine resulting in T cell hyporesponsiveness in HIV-1-infected individuals [37], [38]. Interestingly, high PD-L1 expression on neutrophils correlated with reduced CD3ζ expression on T cells (Fig. 4C) as well as with increased levels of arginase-1 in plasma (Fig. 5A). Collectively, this data suggests that high PD-L1 expression on neutrophils correlates with the dysregulation of T cell function in HIV-1-infected subjects.

### Neutrophil PD-L1 expression correlates with elevated levels of markers of neutrophil degranulation *in vivo*

Although the etiology of LDNs is unclear, some studies indicate that the LDN phenotype is acquired following neutrophil degranulation resulting in co-segregation with the PBMC fraction on density gradient [6], [9], [10]. The content of neutrophil primary, secondary, and tertiary granules is released in response to activation of neutrophils at the site of infection and inflammation and contributes to the creation of an antimicrobial milieu at the inflammatory site [1]. Plasma levels of neutrophil granule proteins myeloperoxidase (MPO), neutrophil gelatinase-associated lipocalin (NGAL), and arginase-1 are significantly higher in HIV-1-infected patients than in uninfected volunteers (E.S.H., Z.H., unpublished data). Importantly, increased PD-L1 expression on neutrophils in whole blood correlated with plasma levels of neutrophil degranulation markers arginase-1, MPO, and NGAL (*p* = 0.05, 0.002, and 0.005, respectively; Fig. 5A–C). In addition, circulating neutrophil PD-L1 expression in viremic ART-naive HIV-1-infected patients directly correlated with plasma levels of G-CSF (R = 0.5; *p = *0.05), GM-CSF (R = 0.7; *p = *0.02), and monocyte chemoattractant protein-1 (MCP-1/CCL2) (R = 0.7; *p = *0.003; Figure 5D–F). These proteins have been demonstrated to play important roles in the recruitment, activation, and chemoattraction of neutrophils to the inflamed tissue. Collectively, these results indicate that elevated PD-L1 expression on neutrophils is closely associated with the production of factors involved in neutrophil recruitment and increased rate of neutrophil degranulation *in vivo* [1], [2].

## Discussion

The study presented here reveals four major findings: (i) neutrophils in blood of HIV-1-infected individuals express elevated level of PD-L1; (ii) the level of neutrophil PD-L1 expression correlates with the expression of PD-1 on CD8^+^ and CD4^+^ T cells and CD57 on CD4^+^ T cells and decreases following ART; (iii) PD-L1 on neutrophils is induced by multiple stimuli including HIV-1, TLR-7/8 ligand R848, IFNα, and LPS; and (iv) PD-L1/PD-1 pathway contributes to the suppression of T cell function by neutrophils. Taken together, these findings are consistent with a hypothesis that HIV-1 infection and ongoing microbial translocation induce neutrophils with an immunosuppressive activity that significantly contributes to the suppression of T cell function in HIV-1-infection. This novel mechanism of immune suppression mediated by neutrophils may alter our understanding of HIV-1 pathogenesis and result in a design of novel therapies targeting the loss of immune function in HIV-1-infected individuals.

The results presented here are consistent with previous studies demonstrating the suppressive activity of activated neutrophils [2], [6]–[9] [10]. Since neutrophils readily interact with T cells in inflamed tissue and secondary lymphoid organs [2]–[4], [7], [39], [40], neutrophil PD-L1 is likely to significantly contribute to the PD-1-mediated suppression of T cell function. The data presented here are strongly supported by a report published at the time of submission of this manuscript demonstrating that IFNγ-stimulated neutrophils suppress T cell proliferation via the expression of PD-L1 [41]. Interestingly, PD-L1 expression is significantly elevated in the suppressive CD16^+^CD62L^low^ subpopulation of neutrophils that is induced in human volunteers following injection of a low dose of LPS [7], [41]. Neutrophil-mediated immune suppression can be highly beneficial in acute sepsis [42] and acute viral infection [43], [44] where it limits the damage caused by the host's inflammatory response and prevents excessive tissue damage. However, it exerts a detrimental effect in the context of prolonged immune activation such as chronic viral infections and cancer by inducing long-term attenuation of T cell functionality [1], [5], [7]. It is likely that immune suppression mediated by PD-L1 on neutrophils plays a role in the pathogenesis of other viral and bacterial infections. Increased expression of PD-L1 on neutrophils was recently described in patients with active tuberculosis [45]. Future *in vivo* studies utilizing murine and/or simian models will be critical to delineate the significance of neutrophil mediated suppression of T cell function via the PD-L1/PD-1 pathway.

The results presented here are consistent with the study of Volbrecht *et al*. demonstrating an expansion of CD15^+^ CD33^+/dim^ CD11b^+^ G-MDSC population in HIV-1 infection [27] and with the studies by Cloke *et al.* describing a population of activated low-density granulocytes/neutrophils (LDNs) in PBMCs of HIV-1-infected patients [28], [46]. Similarly, studies in cancer patients showed an expansion of CD14^−^CD15^+^CD11b^+^ G-MDSC population with the ability to inhibit T cell function via ROS- and arginase-1-dependent mechanisms [6], [9], [10], [47]. Distinct subpopulations of circulating neutrophils were identified by several studies; however, the phenotype and physiological function of these populations remain elusive [48]. In contrast to several previous studies in cancer but consistent with a recent study by de Kleijn *et al.* [41], we demonstrate that certain phenotypic changes, such as elevated PD-L1 expression, occur in entire circulating neutrophil population and are not restricted to the subpopulation of LDN/G-MDSC neutrophils co-segregating with PBMCs. Although the relative contribution of LDNs versus blood neutrophils to immune regulation in HIV infection is unclear, we propose that the PD-L1-mediated suppression of T cell function is not restricted to LDNs and can be mediated by a significant part of the entire circulating neutrophil population. We propose that neutrophils represent a highly sensitive sensor of chronic inflammation and function as a negative feedback mechanism curbing the detrimental impact of systemic immune activation. Association between PD-L1^high^ phenotype and systemic neutrophil activation and degranulation *in vivo* is supported by correlations with plasma levels of markers of neutrophil degranulation (MPO, NGAL, and arginase-1), production of factors involved in neutrophil recruitment and activation, and production of ROS (Fig. 5). These results are in agreement with a recent study suggesting that circulating neutrophils in HIV-1-infected patients become progressively more activated and degranulated with increased disease severity [28]. Thus, elevated PD-L1 expression may represent an early stage of neutrophil activation preceding the acquisition of LDN/G-MDSC phenotype [49].

Neutrophils are highly sensitive to experimental manipulation and become easily activated during preparation [6], [7]. In this study we utilized techniques minimizing the stress associated with neutrophil enrichment such as the use of isotonic red blood cell lysis buffer. Collectively, these techniques resulted in a neutrophil population that was highly viable at 24 hours of *in vitro* culture (Figure S5). Experiments elucidating the role of PD-L1 in T cell suppression were performed in the presence of ROS scavengers. Production of ROS appears to be a major mechanism of neutrophil and G-MDSC-mediated suppression of T cell function [6]–[8]. Importantly, PD-L1-mediated suppression appears to function independently of ROS and is contingent on elevated PD-1 expression on T cells. Release of arginase-1 from neutrophils, depletion of arginine, and subsequent downregulation of TCR CD3ζ chain represent another potential mechanism of neutrophil-mediated immune suppression [9], [10], [37], [47], [50]. Supporting this mechanism is the data demonstrating a correlation between PD-L1 expression on neutrophils, elevated concentration of arginase-1 in plasma (Fig. 5A), and reduced CD3ζ expression on T cells (Fig. 4C). This data is in agreement with a report by Cloke *et al.* demonstrating that the level of arginase-1 activity in PBMCs of HIV-seropositive patients increases with disease severity, inversely correlates with CD3ζ expression on T cells, and that the main source of arginase-1 are neutrophils co-purifying in the PBMC fraction [28], [37], [46].

Depletion of suppressive CD15^+^ neutrophil population co-segregating in the PBMC fraction results in a reversal of suppression and increased cytokine production by T cells (Fig. 2). Multiple studies have described limited production of IFNγ, TNFα, IL-2 and other factors by antigen-specific T cells from viremic HIV-1 patients [12]. Such studies may be confounded by the presence of suppressive LDNs limiting the functionality of T cells in *in vitro* assay.

The upregulation of neutrophil PD-L1 expression in HIV-1-infected patients is likely caused by a combined effect of several factors. We show that inactivated HIV-1 virions and a TLR-7/8 ligand directly upregulate neutrophil PD-L1 (Fig. 3). PD-L1 expression is higher on neutrophils obtained from viremic patients and becomes significantly reduced following the initiation of ART (Fig. 1). This data suggests that PD-L1 is directly induced by the virus, consistent with previous reports in monocytes and dendritic cells [25], [26]. However, other factors may contribute to this process. HIV-1 infection is associated with an extensive damage to the intestinal mucosal barrier and ensuing translocation of microbial products including LPS [30]. LPS cognate receptor TLR-4 is expressed at high levels on the surface of neutrophils and mediates the recognition of gram-negative bacteria [51]. Since LPS upregulates neutrophil PD-L1 (Fig. 3), microbial translocation may directly contribute to the induction of PD-L1^high^ neutrophil phenotype. This view is strongly supported by a recent report demonstrating that an injection of low dose of LPS into the circulation of human volunteers causes an induction of a suppressive neutrophil population inhibiting T cell function via PD-L1/PD-1 [7], [41]. Boasso *et al.* have shown that HIV-1-induced PD-L1 upregulation on monocytes is in part dependent on IFNα [29]. Consistent with this report, we show that blocking IFNα receptor partially blocked PD-L1 upregulation (Figure S2B). Importantly, high levels of IFNα are detected in both plasma and lymphoid tissues during different stages of HIV-1 infection [52], [53] and therefore may contribute to the increased levels of PD-L1 on neutrophils.

The novel model of immune suppression mediated by neutrophils via the PD-L1/PD-1 pathway presented in this study enhances our understanding of T cell exhaustion in HIV-1 infection and highlights the need to target immunosuppressive pathways such as PD-L1/PD-1 in future therapeutic approaches in HIV-1-infection. Blocking the activation and suppressor function of neutrophils could improve immune competence in patients with AIDS.

## Materials and Methods

### Ethics statement

All procedures involving human subjects were approved by the Institutional Review Board of the University of Alabama at Birmingham. All participants in this study were adults. Informed consent was obtained from all participants.

### Patient recruitment and cell isolation

Blood was collected from healthy donors (HD) and HIV-1 infected donors using acid citrate dextrose (ACD) collection tubes. 16 HIV-1-infected subjects on antiretroviral therapy (ART) (median HIV-1 viral load = 190 (20–10,700) vRNA copies per ml; median CD4^+^ T cell count = 444 [102–1,385] per µl of blood); and 21 HIV-1-infected subjects off ART therapy (median viral load = 19,900 [71–1,040,000]; median CD4^+^ T cell count = 657 [189–1,763]) were recruited for the purpose of this study. Peripheral blood mononuclear cells (PBMCs) were isolated by density centrifugation using Lymphocyte Separation Media (MP Biomedicals; Solon, OH). Polymorphonuclear cells (PMNs) were isolated by density centrifugation using Ficoll-Paque Premium (GE Healthcare). Briefly, after centrifugation, the mononuclear cell layer was removed and the granulocyte layer was collected and resuspended. The erythrocytes were lysed with isotonic NH_4_Cl erythrocyte lysis buffer (170 mM NH_4_Cl, 10 mM KHCO_3_, 20 µM EDTA, pH 7.3) [7]. This procedure results in ≥95% purity of neutrophils as determined by the expression of CD15 marker. Cells were cultured in RPMI 1640 supplemented with 5% human A/B serum (Atlanta Biologicals; Atlanta, GA), 100 I.U./mL penicillin, 100 µg/mL streptomycin, 2 mM L-glutamine, and 1× minimal essential amino acids (Life Technologies, Grand Island, NY). Positive selection of CD3^+^ T cells and depletion of CD14^+^ monocytes were performed using anti-CD3 FlowComp or anti-CD14 Dynabeads magnetic microbeads, respectively (Life Technologies; Grand Island, NY).

### Materials

All cell culture reagents were obtained from Mediatech Inc. (Manassas, VA), unless indicated otherwise. Antibodies, beads and columns for cell purification were obtained from Life Technologies/Invitrogen (Grand Island, NY). Antibodies for flow cytometry were purchased from eBioscience (San Diego, CA), unless listed otherwise. HIV-1 MN (X4-tropic) virus inactivated with aldrithiol-2 (AT-2) and control microvesicles from uninfected cell cultures were kindly provided by AIDS and Cancer Virus Program, SAIC Frederick, Inc., National Cancer Institute (Frederick, MD). HIV-1 Consensus B Gag specific peptide pool (15-mers) was provided by the NIH AIDS Research and Reference Reagent Program (Germantown, MD). Plasma levels of myeloperoxidase (MPO), arginase-1, and neutrophil gelatinase- associated lipocalin (NGAL) were determined using ELISA according to the manufacturer's protocol (Hycult Biotech, Uden, The Netherlands).

### Analysis of cell populations

To analyze the phenotype of neutrophils and other cells in blood, 50 µL of freshly obtained whole blood (within 2 hours past blood draw), was incubated for 20 minutes with indicated antibodies, lysed with erythrocyte lysis buffer, and analyzed on LSRII (BD Biosciences, San Diego, CA). PBMCs were blocked in PBS complemented with 10% human A/B serum (Atlanta Biologicals; Atlanta, GA) for 20 min prior to staining in staining buffer (PBS containing 2% FBS). To analyze the expression of CD3ζ-PE, T cells were stained with CD3-efluor450 and CD8-PerCP-Cy5.5, permeabilized, and stained with CD3ζ-PE antibody (clone 6B10.2). Intracellular cytokine staining was performed using the Cytofix/Cytoperm Fixation/Permeabilization Solution Kit (BD Biosciences; San Jose, CA). Briefly, cytokine production was stimulated with 2 µg/mL HIV-1 consensus B Gag specific peptide pool (15-mers) (NIH AIDS Research and Reference Reagent Program; Germantown, MD) for 24 hours in PBMCs or PBMCs depleted of CD15^+^ neutrophils. T cells were stained with CD3-efluor450 and CD8-PerCP-Cy5.5, permeabilized, and stained with IFNγ-APC.

### *In vitro* stimulation of neutrophils

Whole blood, PBMCs, or PMNs were cultured with human interferon-α (IFNα) (1,000 U/ml, Alpha A/D hybrid, #11200, PBL Interferon Source; Piscataway, NJ), R848 (5 µg/ml, InvivoGen, San Diego, CA), AT-2 HIV-1_MN_ (3–1,500 ng/ml p24 capsid equivalent), control microvesicles, or lipopolysaccharide (LPS) (100 ng/ml, *Escherichia coli* 0111:B4; Sigma). Cells were then blocked in PBS with 10% human serum for 20 min at 4°C, resuspended in 100 µl of master mix containing staining buffer (PBS with 2% FBS) and antibodies: CD15-FITC (Biolegend), CD14- PerCP-Cy5.5, and PD-L1-APC (Biolegend). Media for experiments using AT-2_MN_ was supplemented with FBS instead of human serum to avoid potential blocking effects of human serum [54]. Blocking of human IFNα was performed by pre-incubating PBMCs or PMNs with 5 µg/ml anti-IFNAR (clone MMHAR-2; Invitrogen) for 30 min before addition of AT-2 HIV or R848.

### Neutrophil/T cell suppression assay

Purified T cells were stimulated with plate bound anti-CD3 (2 µg/mL) and soluble anti-CD28 (2 µg/mL) antibodies for 24 hours. CD15^+^ Neutrophils were co-cultured with CD3^+^ T cells at a 5∶1 ratio in media supplemented with catalase (1000 U/mL) and superoxide dismutase (200 U/mL; Sigma) to neutralize reactive oxygen species. PD-L1 dependent T cell suppression was neutralized by addition of anti-PD-L1 antibody (clone 29E.2A3, Biolegend); isotype control antibody served as a control treatment (IgG2b; MCP-11; Biolegend). IFNγ ELISA was performed in cell culture media following manufacturer's protocol (eBioscience, San Diego, CA).

### Statistical analysis

Data was analyzed using Student's *t*-test, Mann-Whitney rank sum test, and Wilcoxon signed-rank test as appropriate. Paired *t*-test was used on populations passing the Kolmogorov-Smirnov normality distribution test. Correlations were performed using Spearman rank order test or by Pearson product-moment correlation test for populations that passed D'Agostino & Pearson omnibus normality test. A standard level of statistical significance α = 0.05 was used; all reported *p*-values are two-sided. GraphPad Prism 5 (GraphPad Software Inc., LaJolla, CA) statistical and graphing software packages were used.

## Supporting Information

Figure S1 **Phenotypic characterization of neutrophil and monocyte populations of HIV-1-infected individual.** Expression of various markers on CD14^+^ monocytes and CD15^+^ neutrophils in whole blood of HIV-1-infected subjects was assessed.
(DOCX)

Figure S2 **A**) **Analysis of markers of activation on CD15^+^ neutrophils in PBMCs.** PBMCs were incubated overnight with vehicle (PBS), R848 (5 µg/ml), AT-2 (1500 ng/ml p24 equivalent), IFNα (1000 U/ml), or LPS (100 ng/ml) for 24 hours. PBMCs were then stained and neutrophils were selected as CD15^+^. Data represented as relative to PBS incubated controls (n = 3). B) Effect of anti-IFNA receptor-blocking antibody on induction of PD-L1. PBMCs were stimulated with AT-2 HIV (1,500 ng/mL p24) or R848 (5 µg/ml) in the presence or absence of anti-IFNA receptor blocking antibody for 24 hours. Data are presented as percentage of MFI in the presence of the IFNA receptor-blocking antibody compared to control (N = 4). C) Induction of PD-L1 by LPS is blocked in the presence of polymyxin B. Representative histogram of PD-L1 expression on CD15^+^ neutrophils following incubation of whole blood for 24 hrs with LPS alone (100 ng/mL) or LPS in the presence of polymyxin B (PB; 5 µg/ml).
(DOCX)

Figure S3 **Gating strategy for the analysis of T cell expression of CD57.** A) Strategy for gating T cells. B) Analysis of CD57 expression on CD4^+^ and CD8^+^ T cells, isotype control is shown in grey.
(DOCX)

Figure S4 **Correlation of PD-L1 expression on monocytes with the expression of PD-1 and CD57 on T cells.** CD3^+^CD8^+^ and CD3^+^CD4^+^ T cells from PBMCs were stained for PD-1 (A) and CD57 (B). A) Correlation analysis of PD-1 expression on CD8^+^ and CD4^+^ T cells and PD-L1 expression on monocytes. B) Correlation analysis of CD57^+^ cells as percentage of total CD3^+^CD8^+^ and CD3^+^CD4^+^ T cells with PD-L1 expression on monocytes from the same donors. In all instances, the correlations showed lower statistical significance compared to the correlation with PD-L1 expression on neutrophils.
(DOCX)

Figure S5 **PMN survival analysis.** Isolated PMNs from HIV-1 donors were cultured for the indicated periods of time and stained with Annexin V. Immediately after isolation (Day 0) 0% of the cells stained positive for Annexin V; at 24 hours (Day 1) 6.4% of neutrophils were positive and 48 hours (Day 2) 25.9% of neutrophils were Annexin V positive.
(DOCX)
