# Supplementary figures and images for: Immune Suppression by Neutrophils in HIV-1 Infection: Role of PD-L1/PD-1 Pathway
Source: PLoS Pathog. 2014 Mar 13;10(3):e1003993. doi: 10.1371/journal.ppat.1003993 (PMC3953441; doi:10.1371/journal.ppat.1003993)

**A**

**CD11b**

**CD62L**

**HLA-DR**

**CD114**

**CD80**

**CD124**

**CD16**

**CD115**

**CD86**

**PD-L2**

**CD64**

**CD11c**

**DC-SIGN**

**CD116**


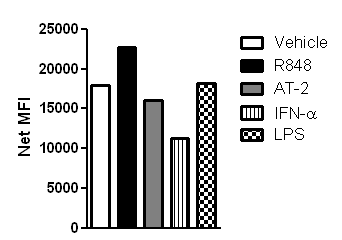

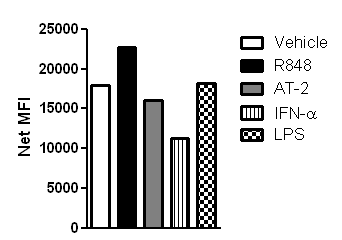

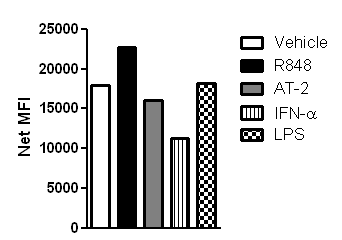

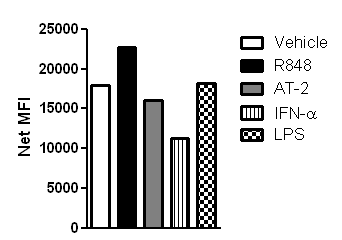

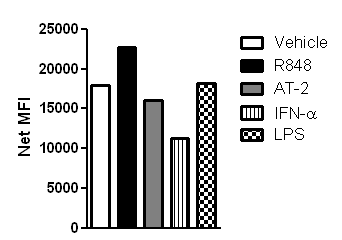


**B C**

Supplement: Figure S2 — A) Analysis of markers of activation on CD15+ neutrophils in PBMCs. PBMCs were incubated overnight with vehicle (PBS), R848 (5 µg/ml), AT-2 (1500 ng/ml p24 equivalent), IFNα (1000 U/ml), or LPS (100 ng/ml) for 24 hours. PBMCs were then stained and neutrophils were selected as CD15+. Data represented as relative to PBS incubated controls (n = 3). B) Effect of anti-IFNA receptor-blocking antibody on induction of PD-L1. PBMCs were stimulated with AT-2 HIV (1,500 ng/mL p24) or R848 (5 µg/ml) in the presence or absence of anti-IFNA receptor blocking antibody for 24 hours. Data are presented as percentage of MFI in the presence of the IFNA receptor-blocking antibody compared to control (N = 4). C) Induction of PD-L1 by LPS is blocked in the presence of polymyxin B. Representative histogram of PD-L1 expression on CD15+ neutrophils following incubation of whole blood for 24 hrs with LPS alone (100 ng/mL) or LPS in the presence of polymyxin B (PB; 5 µg/ml). (DOCX) [file ppat.1003993.s002.docx]
