# Supplementary material for: Immune Suppression by Neutrophils in HIV-1 Infection: Role of PD-L1/PD-1 Pathway
Source: PLoS Pathog. 2014 Mar 13;10(3):e1003993. doi: 10.1371/journal.ppat.1003993 (PMC3953441; doi:10.1371/journal.ppat.1003993)
Supplement: Supplementary file 2 [file ppat.1003993.s001.docx]

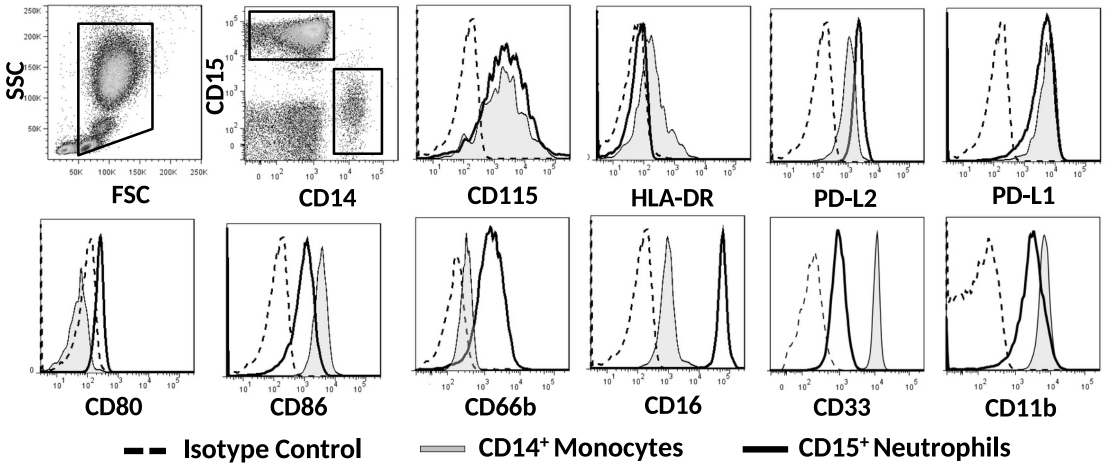
SSC
FSC
CD15
CD14
CD115
HLA-DR
PD-L2
PD-L1
CD80
CD86
CD66b
CD16
CD33
CD11b
Isotype Control
CD14+ Monocytes
CD15+ Neutrophils
