# Supplementary material for: Immune Suppression by Neutrophils in HIV-1 Infection: Role of PD-L1/PD-1 Pathway
Source: PLoS Pathog. 2014 Mar 13;10(3):e1003993. doi: 10.1371/journal.ppat.1003993 (PMC3953441; doi:10.1371/journal.ppat.1003993)
Supplement: Supplementary file 3 [file ppat.1003993.s003.docx]

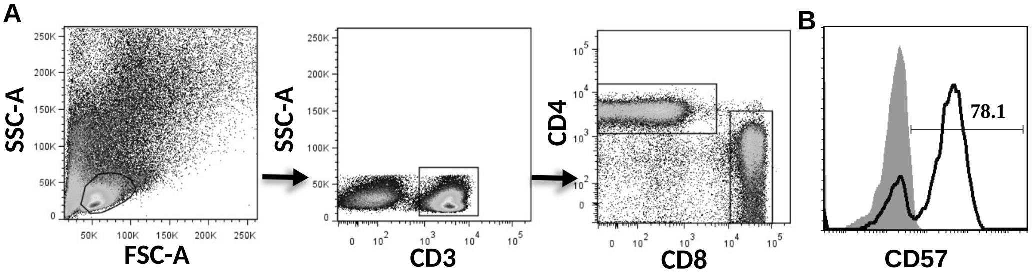

A
SSC-A
FSC-A
SSC-A
CD3
CD4
CD8
B
78.1
CD57
