# Supplementary material for: Immune Suppression by Neutrophils in HIV-1 Infection: Role of PD-L1/PD-1 Pathway
Source: PLoS Pathog. 2014 Mar 13;10(3):e1003993. doi: 10.1371/journal.ppat.1003993 (PMC3953441; doi:10.1371/journal.ppat.1003993)
Supplement: Supplementary file 4 [file ppat.1003993.s004.docx]

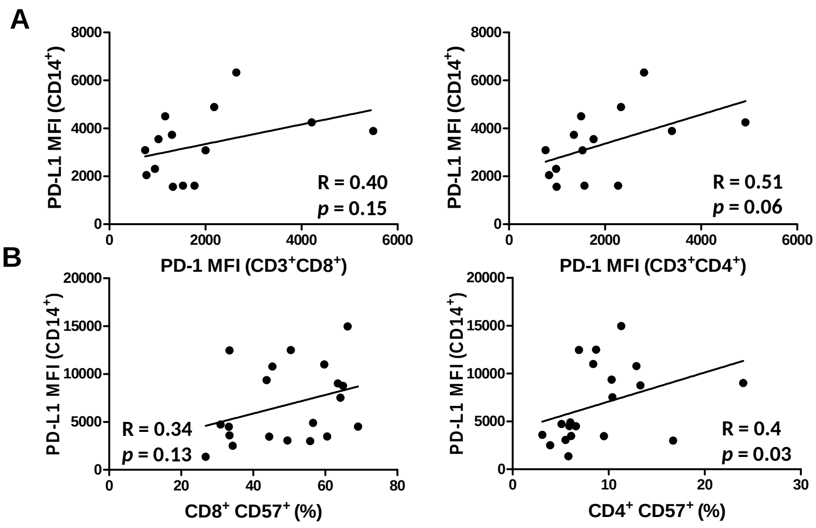
A
B
PD-L1 MFI (CD14+)
PD-1 MFI (CD3+CD8+)
PD-1 MFI (CD3+CD4+)
R = 0.40
p = 0.15
R = 0.51
p = 0.06
CD8+ CD57+ (%)
CD4+ CD57+ (%)
R = 0.34
p = 0.13
R = 0.4
p = 0.03
