# Supplementary material for: Immune Suppression by Neutrophils in HIV-1 Infection: Role of PD-L1/PD-1 Pathway
Source: PLoS Pathog. 2014 Mar 13;10(3):e1003993. doi: 10.1371/journal.ppat.1003993 (PMC3953441; doi:10.1371/journal.ppat.1003993)
Supplement: Supplementary file 5 [file ppat.1003993.s005.docx]

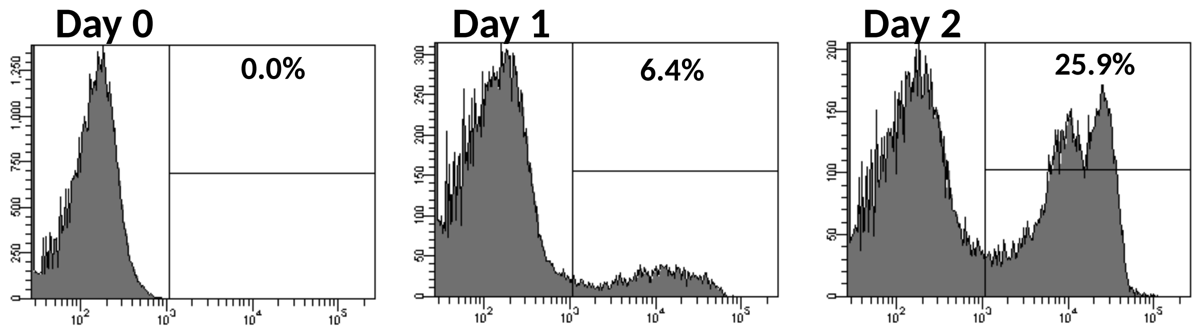
Day 0
0.0%
Day 1
6.4%
Day 2
25.9%
